# Implementation of remote-sensing models to identify post-disaster health facility damage: Comparative approaches to the 2023 earthquake in Turkey

**DOI:** 10.1371/journal.pdig.0001060

**Published:** 2025-10-27

**Authors:** Anu Ramachandran, Akash Yadav, Andrew Schroeder

**Affiliations:** 1 Center for Innovation to Implementation, Veterans Affairs Palo Alto Health Care System, Menlo Park, California, United States of America; 2 Department of Health Policy, Stanford University, Stanford, California, United States of America; 3 Direct Relief, Santa Barbara, California, United States of America; Hadassah Academic College, ISRAEL

## Abstract

Earthquakes and other disasters often cause substantial damage to health facilities, impacting short-term response capacity and long-term health system needs. Identifying health facility damage following disasters is therefore crucial for coordinating response, but ground-based evaluations require substantial time and labor. Artificial intelligence (AI) models trained on satellite imagery can estimate building damage and could be used to generate rapid health facility damage reports. There is little published about methods of generating these estimates, testing real-world accuracy, or exploring error. This study presents a novel method of overlaying model damage outputs with health facility location data to generate health facility damage estimates following the February 2023 earthquake in Turkey. Two models were compared for agreement, accuracy, and errors. Building-level damage estimates were obtained for Model A (Microsoft neural network model), and Model B (Google AI model), and overlaid with health facility location data to identify facilities with significant damage. Model agreement, sensitivity and specificity for damage detection were calculated. A descriptive review of common error sources based on selected satellite imagery was conducted. A spatially aggregated damage estimation, based on proportion of buildings damaged in a 0.125km^2^ area, was also generated and assessed for each model. Twenty-five hospitals, 13 dialysis facilities, and 454 pharmacies were evaluated across three cities. Estimated damage was higher for Model A (10.4%) than Model B (4.3%). Cohen’s kappa was 0.32, indicating fair agreement. Sensitivity was low for both models at 42.9%, while specificity was high (A:93.6%, B:96.8%). Agreement and sensitivity were best for hospitals. Common errors included building identification and underestimation of damage for destroyed buildings. Spatially aggregated damage estimates yielded higher sensitivity (A:71.4%, B:57.1%) and agreement (Cohen’s kappa 0.38). Leveraging remote-sensing models for health facility damage assessment is feasible but currently lacks the sensitivity to replace ground evaluations. Improving building identification, damage detection for destroyed buildings, and spatially aggregating results may improve the performance and utility of these models for use in disaster response settings.

## Author summary

Earthquakes and other disasters can cause significant damage to health facilities. Understanding the scale of impact is important to plan for disaster response efforts and long-term health system rebuilding. Current manual methods of assessing health facility damage, however, can take several weeks to complete. Many research teams have worked to develop artificial intelligence models that use satellite imagery to detect damage to buildings, but there is still limited understanding of how these models perform in real-world settings to identify damage to healthcare facilities. Here, we take two models developed after the February 2023 earthquake in Turkey and overlay their findings with the locations of three types of health facilities: hospitals, dialysis centers, and pharmacies. We examine the accuracy and agreement between the two models and explore sources of error and uncertainty. We found that it was feasible to overlay these data to yield rapid health facility damage reports, but that the sensitivity of the models was low for the health facilities evaluated. We discuss the key sources of error and ways to improve the accuracy and usability of these models for real-world health facility analysis.

## Introduction

Understanding the damage to health facilities after disasters is crucial to understanding health system capacity and informing resource allocation for short and long-term response efforts. These assessments are typically performed by on-site civil engineering teams and are time and resource-intensive to conduct, often taking weeks to months [1–3]. Ground-based facility assessments can be hampered by damage to transport infrastructure, perilous conditions for staff performing surveys, and competing priorities such as search-and-rescue efforts [1,4]. Moreover, certain types of health facilities may not be included in official assessments, such as private clinics, dental offices, and pharmacies, but remain relevant for health system functioning. As disasters become more frequent, improving the efficiency and scope of health facility assessment is critical.

Recent efforts have been made to leverage geospatial data from remote-sensing technologies to develop ways of identifying and classifying building damage from afar [5,6]. When satellite imagery is available after a disaster, computational models can be trained and validated rapidly to calculate building-level or spatially aggregated estimates of damage. To this end, multiple models have been developed and trained following disaster events such as tsunamis, floods, and earthquakes, employing a range of machine learning and deep learning techniques [2,7–11]. Their aim is ultimately to augment ground-based assessments and provide rapid, accurate data on building damage to support response efforts [12–14]. These models, if implemented effectively, could improve post-disaster response capacity by assessing health system infrastructure with fewer resources and time and increasing the scope of facility types that can be included. This would allow disaster response teams to coordinate emergency medical services in the short term and inform the rebuilding of key infrastructure for long-term health system capacity.

While these models have potential to be valuable tools in disaster response, relatively little literature exists on their implementation in the context of evaluating health facilities. Currently there is no standardized method of combining the building damage data they generate with data on health infrastructure. Different models vary substantially in conceptual approach, training data, output parameters, and internal validation mechanisms, and may have different performance characteristics when applied to health facilities. Their accuracy and agreement in the evaluation of health infrastructure, along with the relevant sources of uncertainty when implemented for this purpose, remains largely unknown. Improving our understanding of how these models perform in post-disaster health infrastructure assessment, along with notable sources of error, can improve their utility and guide their implementation.

In this study, we present two methods for overlaying model-generated building damage data with health facility location data to produce rapid and scalable health facility damage reports following the February 2023 earthquake in Turkey. We describe and compare two satellite-imagery based models and the resulting health facility damage reports, including validation against official assessments where available and review of satellite imagery. Finally, we explore data sources, facility-level and model features that may contribute to the accuracy and utility of model-supported health facility damage assessments.

### Region of analysis

On February 6th, 2023, two earthquakes of magnitude 7.8 and 7.7 struck southern Turkey and northern Syria. They were among the strongest earthquakes ever recorded in the region, and resulted in widespread damage, tens of thousands of casualties, and over a million displaced persons [15]. Turkey has a well-developed response capacity organized by the Disaster and Emergency Management Presidency, while Northern Syria is part of an active conflict zone with limited health services. Immediate response efforts were coordinated and deployed, but hampered by adverse weather conditions including freezing temperatures, limited passable roadways, and damage to electrical and water infrastructure [16]. To help support disaster response, two satellite-imagery based models were developed and trained to identify building damage in affected areas. Three cities in Southeastern Turkey were selected for analysis: Antakya, Kahramanmaraş, and İskenderun. All had evidence of significant earthquake-related damage and data available for health facility location and building damage from both models.

## Methods

### Models utilized

A structured overview of key features for each Model is presented in Table 1.

**Table 1:** Overview of Models Utilized for Health Facility Damage Assessment.

	Model A	Model B
**Development team**	Microsoft, UC Berkeley, Defense Innovation Unit, and Planet Labs	Google Research and the UN World Food Programme Innovation Accelerator
**Model type**	Convolutional neural network model	Convolutional neural network model
**Satellite Imagery Utilized**	Planet Labs and Maxar Technologies	Airbus Pleiades and Maxar WorldView
**Building Identification method**	Building polygons defined by Planet Labs and Maxar Technologies	Building centroid points defined by the Open Buildings dataset
**Training data**	xBD dataset, satellite imagery with open-source labelling	Analyst-labelled satellite imagery
**Output description**	Percentage of pixels within a given building footprint polygon that are estimated to be “part of a damaged building”	Four categories of overall building damage: 1. Not damaged, 2. Minor damage, 3. Major damage, and 4. Destroyed
**Threshold for identifying a “damaged building” used in this analysis**	Greater than 40% of a building polygon identified as damaged	Building damage categorized as Major damage or Destroyed

Model A was a collaboration between Microsoft, UC Berkeley, Defense Innovation Unit, and Planet Labs [17]. A convolutional neural network model was initially trained on a large building damage dataset xBD which provides detailed building damage data following different types of disasters [18]. The model was then fine-tuned on each area of interest using a dataset of satellite imagery annotated with labels generated using Microsoft’s open-source labelling tool which allows users to categorizes areas as “Background” “Damaged building” and “Undamaged building”.The model was then run on each area of interest, with output summarized over building footprint datasets from Microsoft and OpenStreetMap. For each building polygon, the percentage of the building footprint considered “part of a damaged building” was computed. Model A’s output consisted of a vector dataset containing polygons of buildings and estimated percent damage per building.

Model B was developed by Google Research and the UN World Food Programme Innovation Accelerator [19]. Satellite imagery was initially annotated by analysts on the Google AI team to include 500 damaged buildings. A two-tower convolutional neural network model was then initially trained on both labelled and unlabeled satellite images. Analysts then spot-checked initial model output to identify inaccuracies and the model was serially retrained on data with these additional labels. Four damage classes were defined: Not damaged, minor damage, major damage, and destroyed. Buildings were identified as centroid points sourced from the Open Buildings dataset. The model predicted the probability that a given building fell into the categories of “major damage” or “destroyed”. For each city, a score threshold was defined at the intersection between precision (defined as how many of the machine predictions are estimated to be correct vs human predictions) and recall (defined as the percentage of human identified damaged buildings were identified by the machine as damaged) and used to determine whether a building in that city was considered damaged. Model B’s output consisted of a dataset of latitude/longitude points representing building centroids, along with a damage class prediction per building (1 for major damage or destroyed, 0 for no damage or minor damage).

**Fig 1:**
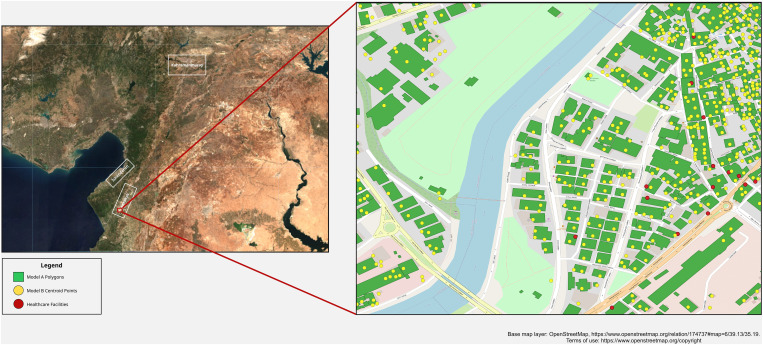
Example output for Models A & B. Model output consisted of Model A Building Polygons (blue) and Model B building centroids (yellow), overlaid with satellite imagery and health facility locations (red). Base map layer: OpenStreetMap, https://www.openstreetmap.org/relation/174737#map=6/39.13/35.19. Terms of use: https://www.openstreetmap.org/copyright.

Example output from each model is shown in Fig 1. To facilitate comparison between the two models given the discrepancy in their conceptual framing (Model A estimating percentage of pixels within a building polygon that were damaged, and Model B estimating the likelihood that a building centroid point fell into the category of major damage or destruction), building damage estimates were simplified into a binary output. A damaged building was defined as Model A estimating that >40% of the pixels in a building polygon were damaged and Model B yielding a damage class prediction of 1, based on the city-specific threshold chosen by the model development team.

### Health facility geocoding

Three categories of health facilities were chosen for analysis: hospitals, dialysis centers, and pharmacies. These categories were chosen based on volume of available location data, access to ground truth damage estimates, representation of different building sizes, and diverse contributions to short and long-term post-disaster health system functioning. Location data for hospitals and pharmacies came from Healthsites.io, an open-source mapping project combining official and crowdsourced health facility locations, provided as latitude-longitude points [20]. Duplicate facilities were excluded. Location data for dialysis facilities were provided by the Turkish Nephrological Institute in the form of facility names and addresses, subsequently geocoded by querying the Google Maps application programming interface to yield latitude-longitude points. Location data was then manually reviewed to ensure accurate mapping.

### Overlay and facility damage estimation

Two methods were developed to generate health facility damage estimates – an individual facility approach and a spatially aggregated approach. Any facilities for which damage data was not available from both models were excluded. All analysis was performed using Python 3.10 and QGIS.

For the individual facility approach, health facility location data was directly overlaid on building damage datasets from Models A and B, using an intersection function and the binary damage definitions described above to yield a list of damaged health facilities in each city for each model. For Model A which utilized building polygons, there were instances where health facility points fell close to but did not fully overlap a building polygon. To account for this, a distance calculation was run using a BallTree algorithm from the sklearn package on Python connecting the centroid of the building polygon to the nearest health facility point. All facilities less than 25 meters from the centroid of a damaged building were filtered and marked as damaged.

For the spatially aggregated approach, all buildings available in the damage datasets were aggregated spatially at a resolution of 0.125 km^2^ for the three regions of interest. The proportion of buildings with significant damage (defined as >40% damage for Model A and a damage class prediction of 1 for Model B) in each grid cell was calculated and grouped into quartiles. Health facility location data was then overlaid on the grid, with each facility taking the damage value of the grid cell into which it fell. Facilities in cells with aggregated damage above the third quartile were considered likely to be damaged.

### Analysis of health facility damage

Health facility damage assessments were organized by facility type and city. The number and percentage of facilities damaged was calculated for each city. Model A and Model B were compared using Cohen’s kappa to determine agreement overall, for each facility type and each city.

Sensitivity and specificity of each model was calculated for dialysis centers and hospitals. Sensitivity was defined as the proportion of buildings with true damage (as defined by the gold standard) that were identified as damaged by each model. Specificity was defined as the proportion of truly undamaged buildings identified as undamaged by each model. The gold standard of damage assessment for dialysis centers was based on ground truth data provided by the Turkish Nephrological Institute. Hospital damage was determined by consensus between two personnel reviewing satellite imagery, categorizing each hospital as minor/no damage, major/total damage, or unable to determine. Given the number, variability, and private ownership structure of pharmacies in Turkey, we were unable to obtain ground truth data regarding the damage status of pharmacies in our regions of interest.

### Exploration of error sources

Review of available satellite imagery (sourced from Bing and Google maps) was performed by two study personnel for all hospitals, all dialysis centers, and a subset of pharmacies. The purpose of review was to identify and describe patterns of error that may contribute to model utility. For each facility, the geocoded health facility location point was overlaid with satellite imagery, Model A building polygons, and Model B building centroid points for the local area. Error identification was based on consensus between the two study personnel, and was classified into one of three categories: geocoding-related (when the geocoded health facility location did not appear to fall on or next to a facility), model building identification (when the polygon or centroid point did not accurately represent the building in question), or model damage classification (when either model mischaracterized the existence of damage to a facility).

### Study sample

Initial facility geocoding yielded a list of 36 hospitals, 17 dialysis facilities, and 568 pharmacies. After excluding duplicate facilities, those located outside of the range of either model, and those without an identifiable facility at the geocoded location, the analytic sample consisted of 25 hospitals, 13 dialysis facilities, and 454 pharmacies.

## Results

### Damage estimates

Damage assessments by region and facility type are summarized in Table 2. Model A categorized 20% of hospitals, 0% of dialysis centers, and 10.1% of pharmacies as damaged, while Model B identified 12% of hospitals, 7.7% of dialysis facilities, and 3.7% of pharmacies as damaged. Pooled estimated damage across all facility types was higher for Model A (10.4%) than for Model B (4.3%).

**Table 2:** Damage classification by Region and Facility Type.

Table One. Damage Estimates, by Facility Type, for Model A & Model B.								
	Hospitals		Dialysis Facilities		Pharmacies		Total	
	Model A	Model B	Model A	Model B	Model A	Model B	Model A	Model B
**Damaged**	5 (20.0%)	3 (12.0%)	0 (0%)	1 (7.7%)	46 (10.1%)	17 (3.7%)	51 (10.4%)	21 (4.3%)
**Undamaged**	20 (80.0%)	22 (88.0%)	13 (100%)	12 (92.3%)	408 (89.9%)	437 (96.3%)	441 (89.6%)	471 (95.7%)
**Total**	25 (100%)		13 (100%)		454 (100%)		492 (100%)	

Based on individual facility model overlay.

### Model agreement and accuracy

For individual facility analysis, overall agreement between the two models was fair, with a pooled Cohen’s kappa of 0.32 (95% CI 0.18-0.46). Notable variability was present by facility type and location - Cohen’s kappa was 0.41 for hospitals, 0 for dialysis centers, and 0.31 for pharmacies, and 0.36 for Antakya, 0.26 for Kahramanmaraş, and 0.05 for Iskenderun. Among hospitals and dialysis centers (N = 38), sensitivity for damage identification was low and the same for Model A and Model B, 42.86% (95% CI 9.90% to 81.59%). Specificity was high and similar between models, 93.55% for Model A (95% CI 78.58% to 99.21%) and 96.77% for Model B (95% CI 83.30% to 99.92%). Sensitivity was higher for hospitals (100% for Model A and 66.67% for Model B) than for dialysis facilities (0% for Model A and 25% for Model B).

### Error exploration

A total of 25 hospitals, 13 dialysis centers, and 12 pharmacies underwent satellite imagery review.. Geocoding errors were identified in 4 (8.0%) reviewed facilities, with 3 cases caused by a geocoded health facility point fell just outside a building, and one case where multiple pharmacy facility points were clustered in a small street, making identification of each individual facility difficult.

Model A building polygon errors occurred in 13 (26.0%), all of which were caused by building polygons that combined multiple smaller buildings into a single polygon with a single damage estimate. Model B centroid point errors in 9 (18.0%), 8 of which were caused by multiple centroid points being assigned to a single, often larger, building, and one case where the centroid point fell outside of the building in question. Identification of damage was considered inaccurate in 11 cases (22.0%) 6 of which were buildings that appeared completely destroyed with substantial rubble that one or both models failed to categorize as damaged.

### Aggregated analysis

**Fig 2:**
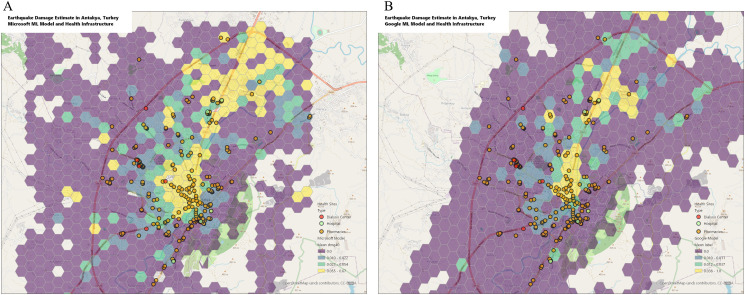
Spatially aggregated damage estimates for Antakya. Each hexagon represents 0.125 km^2^. Hexagons are color-coded by quartile of damage for all buildings in the specified area. Health facility locations are overlaid, color-coded by facility type. Base map layer: OpenStreetMap, https://www.openstreetmap.org/relation/174737#map=6/39.13/35.19. Terms of use: https://www.openstreetmap.org/copyright.

Spatial aggregation at the 0.125 km^2^ level yielded grid cells containing a mean of 36 buildings in the Model A and 98 buildings in Model B, indicating significant differences in the ways that building footprints were identified. Spatially aggregated damage estimates for Antakya, categorized by quartile, are demonstrated in Fig 2. Compared to individual facility estimates, aggregated analysis categorized a larger proportion of facilities as likely to be damaged. Model A identified 24.0% of hospitals, 23.1% of dialysis facilities, and 22.9% of pharmacies, while Model B identified 32.0% of hospitals, 15.4% of dialysis facilities, and 22.7% of pharmacies. Overall model agreement was slightly higher (Cohen’s kappa of 0.38), as was sensitivity for hospitals and dialysis facilities (Model A:71.4%, 95%CI 29.0% to 96.3% Model B: 57.14%, 95% CI 18.4% to 90.1%). Specificity was reduced compared to the individual facility models (Model A: 87.1%, 95% CI 70.2% to 96.4%, Model B: 83.9% 95% CI 66.3% to 94.6%).

## Discussion

This study explores the implementation of remote-sensing model approaches to a key component of disaster response, the identification of health facility damage. It demonstrates the feasibility of overlaying health facility location data with satellite-imagery based building damage models to generate rapid and scalable health facility damage reports after a disaster. When comparing two machine-learning models with similar aims, agreement was generally fair to moderate and varied by facility type and location. When used at the level of individual health facilities, accuracy was best for identifying damage to hospitals, but neither model demonstrated adequate sensitivity to represent a meaningful alternative to ground-based reporting systems. With an alternative spatially aggregated approach to damage estimation, correlation between models and overall sensitivity improved, while specificity decreased.

Rapid estimates of health facility damage can offer substantial value in the context of early disaster response. An initial practical implementation of the models evaluated here could include a rapid spatially-aggregated building damage assessment to identify neighborhoods with both high levels of building damage and high density of health facilities. These will likely represent areas where demand for acute health services is likely to exceed capacity and guide the deployment of short-term resources such as temporary medical facilities or a load-sharing program to redistribute patients to surrounding areas where health facilities are likely to be intact. Continued engagement between model developers and end-users including disaster response agencies is crucial to ensure that model development and outputs are in line with practical implementation needs.

### Error patterns

Our analysis yielded insight into several domains of error that could contribute to the accuracy and utility of remote-sensing models when used to evaluate health facilities. First, maximizing the quality and reliability of health facility location data is crucial to being able to apply these methods. Open-source repositories of health facility location data such as healthsites.io represent many facility types and can be accessed rapidly for use across different disaster situations. Care should be taken to check for duplicate facilities, as were identified in 5 of the 36 initial hospitals listed, and to cross-check with alternative or official sources of health facility data where available. Integrating health facility location data from multiple sources with routine quality checks will greatly facilitate the speed, accuracy, and relevance of the damage estimation outputs. Model agreement and sensitivity was higher for hospitals compared to dialysis centers, which likely reflects their larger size and relative separation from surrounding buildings. The variability in model agreement seen by city could be a function of overall damage, with Antakya having the most overall damage which may improve model coherence based on the improved likelihood of detecting any given damaged building. Alternatively, there may be systematic differences in urban structure which impede model coherence, given differences in building size, arrangement and density. Urban structure variances may also affect the training data used for each model.

Model A polygons were error-prone in areas of higher building density, often combining multiple small buildings into a single polygon and therefore a single damage estimation. This led to inaccurate damage estimations when a damaged building was one part of a larger polygon, thus diluting the damage estimation across the aggregated building polygon footprint. For larger buildings such as hospitals, polygon estimations were more accurate than Model B centroids in the sample of buildings that underwent satellite imagery review. Model B centroid points performed well at identifying small, clustered facilities as separate structures (such as multiple small pharmacies), but became error-prone in assessment of larger facilities such as hospitals, where multiple building centroid points would be assigned to a single large structure. This could result in overestimation of damage when a small portion of a larger facility (such as one wing of a hospital) was damaged, with Model B assigning a centroid point to a single wing and therefore considering the entire building damaged. Accuracy of building identifiers are partly attributable to the sourcing and processing of satellite imagery – Google satellite imagery is typically preprocessed to improve alignment and accuracy of centroid points, for example. Improving the availability, alignment, and interoperability between different sources of satellite imagery would facilitate model comparison and implementation.

An important theme in damage estimation error in both models was their handling of buildings that were completely destroyed, or which appeared indistinguishable from rubble on the satellite imagery review. Six of the 11 inaccuracies in building damage estimation among the reviewed sample were caused by models failing to identify buildings that were completely collapsed, leading to under-estimation of damage and reduction in sensitivity among both models. While this may be partly attributable to variability in satellite imagery source or timing, focused training around fully destroyed buildings and resulting rubble is likely to substantially improve the sensitivity of both models evaluated.

### Aggregated vs building-level analysis

Aggregated estimates of building damage offer certain benefits, including relatively rapid analysis, incorporation of a larger number of buildings for estimating damage, and avoidance of the building-level error sources noted above. One major caveat is that these approaches can only estimate the probability that a given facility is in a heavily damaged area, rather than providing an estimate of damage to the facility itself. Within the context of disaster response, aggregate estimates could serve an important role in developing a rapid global assessment of overall health system damage. They can also provide preliminary risk stratification to focus and inform subsequent efforts such as ground-based evaluations, analyst review of satellite imagery, or optimizing training data for subsequent facility-level models.

### Limitations to consider

As the first analysis to overlay and implement these data for the purpose of health facility damage assessment, multiple best-estimate definitions were employed, including a 25 meter radius between building centroids and health facility locations, a cutoff of 40% for constituting significant building damage, and using the highest quartile of spatially aggregated areas of damage. While these are reasonable initial estimates, more work is needed to identify and validate appropriate cut-offs for each across different models and contexts, highlighting the broader need for prospective frameworks for remote-sensing health facility damage estimation, which must be a focus of future research. This should include sensitivity analyses with large building datasets testing different damage thresholds for health facilities, as well as external validation to compare damage thresholds from model outputs to operational reporting on health facility functional capacity, where available. Health facilities comprise a wide array of building types, from large hospital complexes to stand-alone clinic buildings and small pharmacies that take up a small portion of a larger commercial structure. Location data on health facilities can vary in quantity and quality, and user-generated location data may not be up to date or accurate, particularly in the case of smaller commercial enterprises such as pharmacies. In a setting with both public and private health infrastructure, official assessments may be more suited to cover public, government-operated facilities such as public hospitals, or facilities with national-level oversight and integration such as dialysis centers.

Lack of ground-truth data for smaller or privately-owned health facilities limits the extent to which we can evaluate the accuracy of these models in this context, particularly as pharmacies represent the majority of health facilities evaluated. As privately-owned and operated entities, high-quality regional data on the operational status of these facilities following the earthquake was not available. This is reflected in the relatively wide confidence intervals for sensitivity and specificity estimates, which were only available for hospitals and pharmacies. While our findings demonstrate useful trends to inform implementation approaches, further analysis with larger datasets is necessary to fully establish the accuracy of these models in practice, particularly to address their ability to identify damage to pharmacies and other small health facilities. Future research should prioritize increasing the scale and scope of ground-based validation efforts for different health facility types. This will include focusing on disaster areas for which health facility operational data is available for pharmacies to improve the external validity of these models. Building damage alone is also likely not sufficient to assess the capacity of a health system in the wake of a disaster and will need to be combined with data on power outages, availability of medications and equipment, and workforce shortages to provide the most accurate assessment.

The variability in damage assessment between the two models demonstrated here can be traced to several sources, including variation between the satellite imagery sources used to train them, the use of a polygon vs a point for identifying a building, or differences in their internal validation mechanisms. While both models were developed to identify building damage after earthquakes, the conceptual framing of the task differed between them, with Model A aimed at estimating percentage damage by building polygon, and Model B attempting to categorize as major damage vs not, based on city-specific thresholds. These differing approaches may result in meaningful differences in the outputs generated and their interpretation, with important consequences for those attempting to operationalize them.

### Conclusions and considerations for implementation

While a large body of work has been generated to develop, train, and compare relative strengths and weaknesses of remote-sensing models for building damage detection, there is far less literature on their implementation in context for health facility assessment. As the state of the art develops, it is reasonable to expect that these models will improve in their capacity, accuracy, and speed of building damage detection. More effort will be needed, however, in translating their output into accurate, useful, and timely recommendations for public health professionals, along with nuanced yet accessible assessments of uncertainty. Developing a standardized framework for selecting appropriate model approaches, optimizing available satellite imagery and health facility location data, and translating model outputs into actionable recommendations should be a focus of future research.

## Supporting information

S1 Data Facility locations and damage estimates used for analysis. (XLSX)

S1 File Geopackages for Model A utilized in analysis. (ZIP)
